# The non-stop road from concrete to abstract: high concreteness causes the activation of long-range networks

**DOI:** 10.3389/fnhum.2013.00526

**Published:** 2013-09-03

**Authors:** Sabine Weiss, Horst M. Müller

**Affiliations:** ^1^Center of Excellence “Cognitive Interaction Technology”, Bielefeld UniversityBielefeld, Germany; ^2^Faculty of Linguistics and Literary Science, Experimental Neurolinguistics Group, Bielefeld UniversityBielefeld, Germany; ^3^Collaborative Research Center “Alignment in Communication” (SFB 673), Bielefeld UniversityBielefeld, Germany

**Keywords:** theta, beta, gamma, EEG frequency, long-range coherence, language, concreteness, embodiment

## Abstract

Current grounding theories propose that sensory-motor brain systems are not only modulated by the comprehension of concrete but also partly of abstract language. In order to investigate whether concrete or abstract language elicits similar or distinct brain activity, neuronal synchronization patterns were investigated by means of long-range EEG coherence analysis. Participants performed a semantic judgment task with concrete and abstract sentences. EEG coherence between distant electrodes was analyzed in various frequencies before and during sentence processing using a bivariate AR-model with time-varying parameters. The theta frequency band (3–7 Hz) reflected common and different synchronization networks related to working memory processes and memory-related lexico-semantic retrieval during processing of both sentence types. In contrast, the beta1 band (13–18 Hz) showed prominent differences between both sentence types, whereby concrete sentences were associated with higher coherence implicating a more widespread range and intensity of mental simulation processes. The gamma band (35–40 Hz) reflected the sentences' congruency and indicated the more difficult integration of incongruent final nouns into the sentence context. Most importantly, findings support the notion that different cognitive operations during sentence processing are associated with multiple brain oscillations.

## Introduction

Modal theories on cognition propose that language is represented in multiple, distributed cognitive systems and is based on the sensory-motor system underlying perception, action and introspection (inner mental states). In many of these so-called “grounding theories” the body is the focus of interest but besides this, the situated nature of actions, the importance of the experience with the environment and cultural influences are often emphasized too (e.g., Barsalou, 2008). One of the core concepts in grounding theories explaining language comprehension and conceptual knowledge is *mental simulation*, which is commonly defined as the mental reenactment of perceptual, motoric, introspective and affective states during cognitive function (e.g., Barsalou, 2008). Most importantly, it is assumed that mental simulation takes place in the same brain regions activated when one either performs an action such as “grasping” or processes the word “grasping” or a sentence referring to that particular action (Gallese and Lakoff, 2005).

An important question in this context is the relation between mental simulation and the concreteness of linguistic stimuli. Most of the studies on grounded cognition underlying language processing investigated concrete language material such as concrete words or sentences. Only a few dealt with the relation between embodiment, mental simulation and the processing of abstract language, which is a major challenge for grounding theories and mental simulation as their core concept (e.g., Pecher and Zwaan, 2005; Glenberg et al., 2008; Dove, 2011; Scorolli et al., 2011 for an overview; Glenberg and Kaschak, 2002; Wiemer-Hastings and Xu, 2005). This leads to the question whether results based almost exclusively on the investigation of concrete language can also be applied to the processing of abstract language. In other words, do we understand abstract language by performing concrete mental simulations and applying concrete mental concepts? Does the processing of abstract language make use of similar neuronal networks and is it as grounded as the processing of concrete language? In seminal studies it has been shown that concrete, linguistic stimuli have many cognitive processing advantages over abstract ones (e.g., Schwanenflugel and Shoben, 1983; Paivio, 1991; Walker and Hulme, 1999). This concreteness effect can be explained at least partly by “grounding theories” postulating concrete language to be based on activity in multimodal sensory-motor systems (e.g., Barsalou and Wiemer-Hastings, 2005; Glenberg et al., 2008). More specifically, it has been proposed that the processing of concrete concepts is based rather on the perception of physical entities and action or introspective and physical states, respectively, while many abstract concepts are understood on account of the simulation of internal, affective states and objective or contextual situations and processes (Wiemer-Hastings and Xu, 2005). As a consequence, the comprehension of concrete and abstract language should not only be associated with common neuronal activity patterns related to perceptual and motoric processes but also demonstrate significant differences in neuronal activity.

Up to now, there are relatively few neuroimaging studies comparing the neurobiological basis of the comprehension of whole concrete and abstract sentences pointing at partly divergent neuronal connectivity (Holcomb et al., 1999; Just et al., 2004; Tettamanti et al., 2005; Wallentin et al., 2005; Boulenger et al., 2009; Ghio and Tettamanti, 2010; Desai et al., 2011). In the current study neuronal connectivity accompanying the processing of concrete and abstract sentences was investigated by means of EEG coherence analysis. EEG coherence is a linear correlation coefficient, which provides information on the functional relation of two time series across the experimental trials (Rappelsberger and Petsche, 1988; Schack et al., 1995; Nunez et al., 1997; Bressler and Kelso, 2001). High EEG coherence means that the neural activity underlying the recorded signals is functionally coupled during a definite experimental task. Moreover, EEG coherence measures the degree of functional cooperation in a certain time and frequency window. The role of EEG frequencies related to language processing is still a matter of intense debate. Increasing evidence supports the notion that brain oscillations at different frequencies (1–40 Hz) indicate distinct operations during language processing at the word- and sentence level such as working memory or episodic memory processes, syntactic role assignment, lexical processing and violation of semantic congruency (Weiss and Mueller, 2003, 2012; Roehm et al., 2004; Bastiaansen and Hagoort, 2006; Mainy et al., 2008; Penolazzi et al., 2009; Luo et al., 2010 for reviews; Pulvermueller et al., 1997).

The particular focus of this study was to investigate the neuronal cooperation underlying the processing of sentences of different concreteness. Long-range coherence analysis was applied to EEG data recorded during the whole course of sentence processing to assess similarities or differences in neuronal cooperation patterns for concrete compared to abstract sentences. Further, to test an interference of concreteness and the sentences' semantic plausibility, congruent and incongruent sentence versions were constructed. This paradigm was partly comparable to that of Holcomb et al. (1999), which analyzed the N400-ERP component during the processing of congruent and incongruent sentences. They reported an interaction of congruence and concreteness. Specifically, they found that concrete and abstract sentences elicited a different scalp distribution of the N400 only in the incongruent and not in the congruent condition. In their study, the concrete final nouns showed a larger negativity at anterior sites. This means that a supportive context canceled out the concreteness effect. According to Holcomb et al. (1999) opinion, the varying time-courses of the concreteness vs. the congruence effects could account for this finding. The anterior concreteness effect started somewhat later and lasted longer (300–500 to 500–800 ms) than the posterior context effect (150–300 to 300–500 ms). A supportive context may generate a contextual representation prior to the onset of the final noun. Therefore, a semantically appropriate word is effortlessly integrated into the sentence context. When an inappropriate word is presented, participants attempt to integrate this word into the sentence context by using all semantic, multimodal information, which is necessary to make sense of the sentence (Holcomb et al., 1999). As stated above, concrete language provides multimodal sensory-motor information, which is only partly found in abstract language.

The results of Holcomb et al. (1999) suggest the following predictions with regard to the coherence analyses in the current experiment. First, concerning cognitive operations during processing concrete and abstract language mentioned above, both sentence types should not only share common neuronal patterns related to perceptual and motoric processing, but also demonstrate significant differences in neuronal processing. Concrete sentences should exhibit higher and more widespread coherence than abstract sentences due to their multimodal sensory-motor representation. Second, a congruence effect should be found at the final noun. Third, the concreteness effect should be canceled out for the congruent sentences, but should exist in incongruent sentences.

## Materials and methods

### Participants

Twenty-nine monolingual students (14 females) aged 19–28 years (mean 23.5; *SD* = 3.5) with German as their native language participated in the EEG-experiment. They all had inconspicuous audiograms of both ears and reported no history of any significant neurological or psychiatric illness. All of the participants were right-handed, gave written informed consent prior to the measurements and were paid for their participation. Another 101 students participated in the various ratings and the reaction time study described in sections Stimuli and Reaction Time Data.

### Stimuli

#### Construction of concrete and abstract sentences

The critical material analyzed in this study consisted of 100 German sentences randomly presented in four blocks. Half of the critical sentences were constructed as concrete, the other half as abstract sentences. The criteria for controlling concreteness/abstractness were as follows: At first, nouns and verbs used for sentence construction were matched on imageability and concrete-/abstractness in order to use only very concrete and very abstract words for the construction of the sentences. All nouns were matched on imageability and concreteness/abstractness according to studies of Baschek et al. (1977) and Offe et al. (1981). On a bipolar scale (−20 < 0 < +20) the mean score for the concrete nouns was 16.50 ± 0.75 for imageability and 16.20 ± 2.40 for concreteness/abstractness. The abstract nouns had −3.18 ± 4.22 for imageability and −4.76 ± 4.38 for concrete-/abstractness. Concrete and abstract nouns differed significantly for the variables “imageability” and “concreteness/abstractness” (*t*-test, 2*p* = 0.0001).

Further, verbs, which were previously rated on a 7-point scale on imageability and concreteness/abstractness (Berghoff, 2002) were used for the construction of the sentences. Only very concrete nouns (see above) and verbs (mean > 5.0) were used for the construction of the concrete sentences and the opposite for the abstract sentences (mean for verbs < 4.0).

#### Psycholinguistic criteria

An important further step was to match sentences for morpho-syntactic surface structure while attempting to keep the plausibility and the complexity of the concrete and abstract sentences equal. Hence, each condition contained a similar proportion of simple, compound and complex sentences (concrete congruent 12:8:5; concrete incongruent 12:9:4; abstract congruent 12:7:6; abstract incongruent 13:8:4). In addition, the average number of words per sentence, the percentage of verbs used and the percentage of one−, two−, and three-syllabic words were controlled. This was undertaken to control for potential cognitive-linguistic differences between concrete and abstract sentences besides their concreteness. Univariate ANOVAs did not reveal any difference between the average number of words for concrete (10.83 ± 1.83) and abstract (10.83 ± 1.60) sentences. The mean amount of syllables also did not differ between concrete (20.5 ± 3.28) and abstract sentences (20.5 ± 3.53).

#### Cloze probability

Further, the cloze-probability for concrete and abstract sentences was equalized. Cloze probability was assessed by a separate group of 32 participants who listened to the sentences without the final noun and filled in what they thought was the most appropriate final word for each sentence. All sentences had a high cloze probability of at least 80% and the concrete and abstract sentences did not differ in cloze probability (*t*-test, 2*p* = 0.26). These results allow us to construct incongruent sentences by semantically violating half of the concrete and abstract sentences with the final noun. No word generated by any of the participants was used for the construction of the incongruent sentences, thus effectively making the cloze values for the incongruent sentences zero.

#### Final stimuli

From these sentences four lists were formed so that half of each group was semantically congruent, the other half incongruent. This resulted in four different groups of sentences:

- Twenty-five concrete congruent sentences: e.g., “Sie sah gern Tiere und darum ging sie in den Zoo.” (*She liked looking at animals, so she went to the zoo*).
- Twenty-five concrete incongruent sentences: e.g., “Sie sah gern Tiere und darum ging sie in den Schrank.” (*She liked looking at animals, so she went into the cupboard*).
- Twenty-five abstract congruent sentences: e.g., “Sie hatte keine Zeit und darum setzte sie ihm eine Frist.” (*She had no time, so she set him a deadline*).
- Twenty-five abstract incongruent sentences: e.g., “Sie hatte keine Zeit und darum setzte sie ihm eine Gunst.” (*She had no time, so she set him a favor*).

#### Rating study

After the construction of the critical sentences a group of 21 students rated each of the sentences on their concreteness/abstractness and imageability on a 7-point scale (Table 1) to determine whether they fulfill the psycholinguistic criteria postulated in section Psycholinguistic Criteria.

**Table 1:** **Mean rating scores on concreteness/abstractness and imageability of the critical sentences**.

	**Concrete/Abstractness**	**Imageability**
Concrete congruent	6.09 ± 0.39	6.81 ± 0.27
Concrete incongruent	4.86 ± 0.54	5.63 ± 0.61
Abstract congruent	3.36 ± 0.63	3.09 ± 0.78
Abstract incongruent	2.26 ± 0.35	1.86 ± 0.48

Univariate ANOVAs were carried out on the rating scores revealing significant main effects for concreteness [*F*_(1, 98)_ = 710.84; *p* = 0.000], imageability [*F*_(1, 98)_ = 1065.28; *p* = 0.000] and congruency [*F*_(1, 98)_ = 134.90; *p* = 0.000]. No interaction between any of the factors was found. As expected, *post-hoc t*-tests revealed significantly higher concreteness and imagery ratings for the concrete sentences. Congruent sentences were rated as being more concrete and imaginable than incongruent ones (*p* = 0.0001, paired *t*-test, 2-tailed). Concreteness and imagery ratings were highly correlated (Pearson corr., 2-tailed, 0.94; *p* = 0.000) which prompted us to use only the concreteness score for further behavioral and EEG analyses. Thus, very concrete and abstract sentences were obtained and used for further analyses.

#### Articulatory features of stimuli

The final sentence stimuli were spoken by a professional male speaker (radio announcer) and recorded in a sound studio (Bielefeld University). Stimuli were digitized at 44.1 kHz, 16 bit and had a mean articulatory duration of 3902 ± 625 ms (concrete: congruent 4061 ± 718 ms, incongruent 3746 ± 586 ms; abstract: congruent 3867 ± 537 ms, incongruent 3935 ± 605 ms). Most importantly, ANOVA revealed no main effects or interactions for concreteness and congruence on articulatory sentence length {concreteness [*F*_(1, 98)_ = 2.18; *p* = 0.143], congruence [*F*_(1, 98)_ = 1.09; *p* = 0.298], interaction [*F*_(1, 98)_ = 0.889; *p* = 0.348]}.

### Experimental procedure

Participants were seated in a sound-attenuating electrically-shielded chamber and had to listen to the sentences via loudspeakers. They were instructed to listen carefully to the sentences and to respond by pressing a “yes” or “no” button after their decision as to whether the sentence was semantically plausible or not. The response hand was counterbalanced across participants, who were instructed to press the button after they heard a tone signal. The tone signal occurred 1000 ms after each sentence's ending to prevent muscle artifacts, elicited by pressing the button. Fifteen hundred millisecond after the participants' answer the next sentence started automatically. The 100 critical sentences were presented randomly dispersed in four blocks of 25 sentences each. After each block a short break was allowed for the participants' relaxation. An EEG was recorded whilst participants listened to the sentences and performed their semantic judgment task.

### EEG recording and analysis

**Figure 1:**
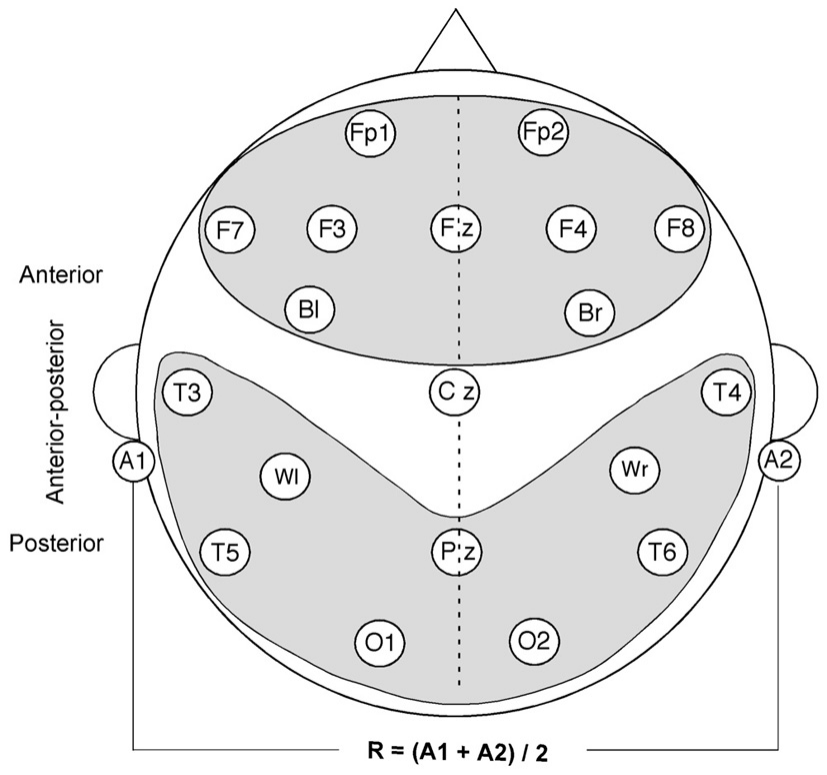
**Scheme of the electrode montage, the reference used (R) and the subdivision into three main domains (anterior, posterior, anterior-posterior) for long-range coherence analysis (gray areas).** Electrodes used for statistical analyses are mapped on the scheme.

The EEG was recorded with a Neuroscan Synamp (with the software Aquire 4.1), from a total of 19 sintered Grass Ag/AgCl electrodes glued to the scalp and 2 electrodes glued to the ear lobes bilaterally. Electrodes were glued to the scalp because participants exhibit fewer muscle artifacts, which are known to influence alpha up to gamma frequencies of the EEG (Rappelsberger, 1998). Fifteen electrodes were placed according to the international 10/20-placement system and 4 additional electrodes (bl, br, wl, wr) were over the Broca and Wernicke areas and their right hemispheric homologs (see Figure 1). The electrooculogram (EOG) was recorded from two electrodes located at the left lateral outer cantus and above the right eye. Data were recorded against the Cz electrode and mathematically re-referenced against the average signals of both earlobes [(A1 + A2)/2].

Electrode impedance did not exceed 8 kOhm and signal band-pass was 0.3–70 Hz. Data were digitally sampled at 256 Hz. After recording, the EEG data were screened for various artifacts (eye blinks, horizontal and vertical eye movements, muscle activities etc.) by visual inspection with Brainvision Analyzer 1 (Brain Products). The spectral analytic evaluation of the data was done with different customized software packages partly established by Schack et al. (1995) and Rappelsberger and Petsche (1988).

Spectral coherence of artifact-free EEG signals was calculated by means of an adaptive fitting procedure for bivariate autoregressive models with a mean least square function as adaptation and with time-varying parameters. Details of the methods are extensively discussed in Schack et al. (1995, 1999). In principle, this method allows a continuous investigation of coherence for each sample point with an arbitrarily high frequency resolution (*p* = 25, adaptation constant = 0.03). For the current study a frequency resolution of 0.5 Hz was chosen and coherence values were obtained approximately every 3.9 ms during sentence processing given the sampling rate (256 Hz) at which the EEG data were sampled.

**Figure 2:**
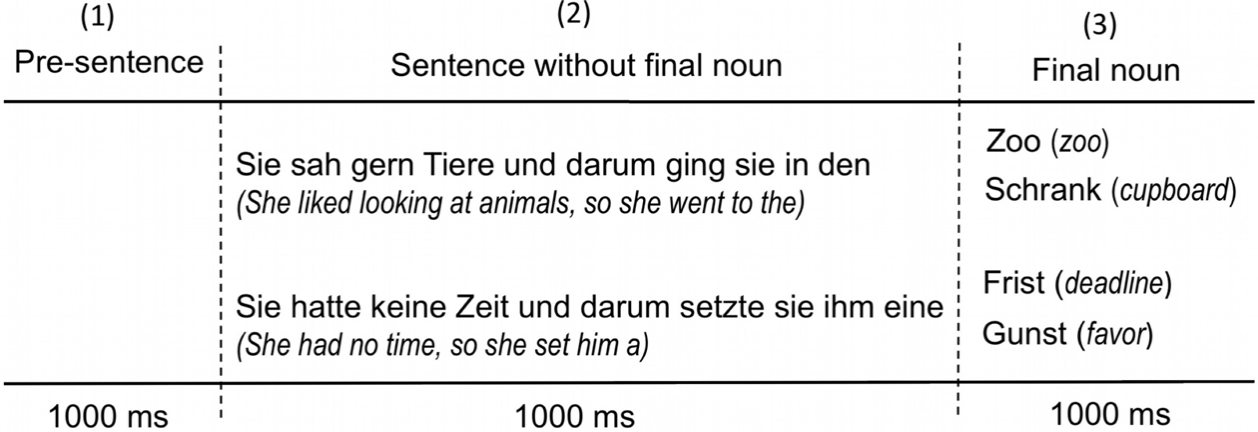
**The three sentence intervals (1, 2, 3) relevant for statistical analysis of the EEG epochs**.

For statistical analysis the following sentence intervals were selected (1) before the beginning of each sentence (pre-sentence: 1000 ms), (2) the sentence without final noun (1000 ms) and (3) the final noun (1000 ms) (see Figure 2).

Cross spectra were computed for each sample point of the critical sentence intervals, for each electrode pair and frequency bin and for each participant time-locked to the beginning of each relevant sentence interval. Adjacent spectral values were averaged to obtain broad frequency bands. We selected frequency bands, which have proven to be strikingly relevant for sentence processing (Roehm et al., 2004; Weiss et al., 2005; Cheung et al., 2009; Bastiaansen et al., 2010; Weiss and Mueller, 2012). Thus, the following frequency bands were used for statistical analyses: theta (3–7 Hz), beta1 (13–18 Hz), gamma1 (29–34 Hz), and gamma2 (35–40 Hz).

Thereafter, coherence was calculated for all “long-range” electrode pairs, which were positioned > 10 cm apart from each other, yielding 140 values per frequency according to the 19 electrode positions analyzed (see Figure 1). The reason for calculating coherence between distant electrodes is that volume conduction may cause a spatial filtering of EEG signals. EEG electrodes, which are closer than 10 cm may receive the average activity of large parts of the same neuronal sources (Nunez and Srinivasan, 2006). Thus, if two closely adjacent electrodes receive activity from the same source and their power increases, then the coherence between the respective electrodes may also increase artificially.

For statistical analyses, coherence values were averaged across time points to receive an average value for each 1000 ms sentence interval. This estimate of mean time coherence is useful because of the high variability of the EEG, which in turn leads to high variability in the adaptive EEG coherence during cognitive processing. In addition, coherence was averaged over different topographical regions (anterior, posterior, and anterior-posterior) (see Figure 1) for each participant for the ANOVAs. This procedure helped us to get an idea of the course behavior of intra- and interhemispheric long-range coherence during sentence processing.

## Results

### Reaction time data

**Figure 3:**
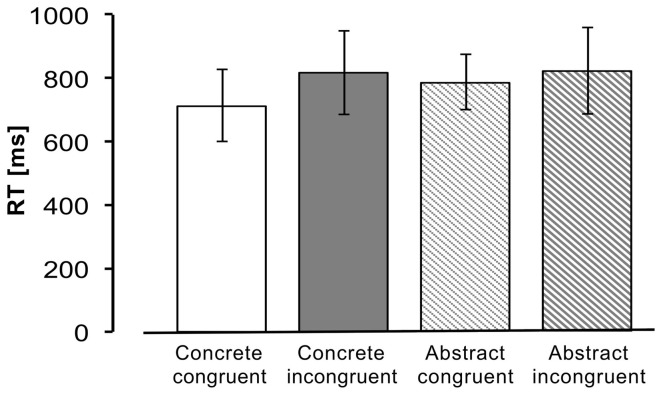
**Bar-plots of the reaction time data for the four different sentence types used in the EEG experiment**.

A reaction time experiment with 26 students proved the well-known concreteness effect for this sentence material. Participants listened to the sentences and had to respond as to whether a sentence was semantically correct or not by pressing a button. Participants showed a mean reaction time of 713.05 ± 113.43 ms for concrete congruent, 817.56 ± 132.33 for concrete incongruent, 787.34 ± 89.30 ms for abstract congruent and 824.18 ± 137.43 for abstract incongruent sentences (Figure 3).

ANOVAs revealed a significant main effect of concreteness [*F*_1(1, 25)_ = 4.41; *p* ≤ 0.04; *F*_2(1, 96)_ = 4.57; *p* ≤ 0.03], congruence [*F*_1(1, 25)_ = 7.107; *p* ≤ 0.01; *F*_2(1, 96)_ = 7.77; *p* ≤ 0.006] and a significant interaction between congruence and concreteness [*F*_1(1, 25)_ = 3.78; *p* ≤ 0.06; *F*_2(1, 96)_ = 5.07; *p* ≤ 0.02] on the reaction time both for subject and item analyses. *Post-hoc* paired *t*-tests revealed that reaction time to concrete compared with abstract sentences is faster for the congruent condition only (*t-test*, *p* ≤ 0.02, 2-tailed). Thus, the concreteness effect was only shown for the congruent condition, whereas in the incongruent condition the reaction time to concrete and abstract sentences did not differ significantly. One explanation concerns the overall lower concreteness of the concrete incongruent sentences suggesting that the semantic implausibility of the sentences cancelled out the concreteness's effect on the reaction time and the error rate. Holcomb et al. (1999) found a similar result on error rates. Moreover, the overall reaction time to congruent sentences was significantly faster than to incongruent ones, which points to a higher processing time for incongruent sentences. The mean percentage of errors associated with the semantic judgment of the sentences was 4.4% across all 100 sentences and all participants. In addition, there was a significant effect of concreteness [*F*_1(1, 25)_ = 26.95; *p* = 0.000; *F*_2(1, 96)_ = 8.29; *p* = 0.005], of congruence [*F*_1(1, 25)_ = 46.13; *p* = 0.000; *F*_2(1, 96)_ = 26.41; *p* = 0.000] and a significant interaction between congruence and concreteness [*F*_1(1, 25)_ = 34.84; *p* ≤ 0.000; *F*_2(1, 96)_ = 7.52; *p* ≤ 0.007] on the error rate. As expected, fewer errors were made during the processing of concrete sentences. However, there was also an effect of congruence on the error rate showing overall fewer errors in the incongruent condition. To summarize, abstract congruent sentences elicited slower reaction times and more errors than concrete ones and reaction time to incongruent sentences was slower than to congruent ones but incongruent sentences elicited fewer errors. Thus, participants were in need of longer decision times for the incongruent sentences but also made fewer errors. These results indicate that the participants' decision processes and task-dependent strategies heavily influence the reaction time.

### EEG data

We applied multivariate analyses to the EEG coherence data of 29 subjects in order to test the main factors and their interactions. Repeated measures ANOVAs were calculated for EEG coherence as a dependent variable with the within-subject factors concreteness (concrete, abstract) and congruence (congruent, incongruent) and the between-subject factors frequency (theta, beta1, gamma1, gamma2) and topography (anterior, posterior, anterior-posterior) for the three relevant sentence intervals (Figure 2). ANOVAs yielded global results on the significance of the main factors and their interactions (Table 2).

**Table 2:** **Repeated measures ANOVAs: significant main effects and interactions for coherence values in the three sentence intervals**.

**Pre-sentence (interval 1)**	**Sentence without final noun (interval 2)**	***F*_(1, 28)_; *p* =**	**Final noun (interval 3)**	***F*_(1, 28)_; *p* =**
–	Concreteness	19.34; 0.000	Congruence	71.37; 0.000
–	Concreteness × Frequency	11.60; 0.000	Congruence × Frequency	22.98; 0.000
–	Concreteness × Topo	7.64; 0.001	Congruence × Topo	16.65; 0.000
–	–	–	Congruence × Frequency × Topo	13.11; 0.000
–	–	–	Concreteness × Topo	6.74; 0.002
–	–	–	Concreteness × Congruence × Topo × Frequency	3.19; 0.004

#### Concreteness and EEG coherence

A main effect of *concreteness* was found in the sentence interval without the final noun (interval 2). The processing of concrete sentences correlated with a significantly higher mean coherence than abstract sentences. Specifically, the theta and beta1 frequency bands showed this effect (concreteness × frequency interaction), particularly at posterior sites (concreteness × topo interaction).

**Figure 4:**
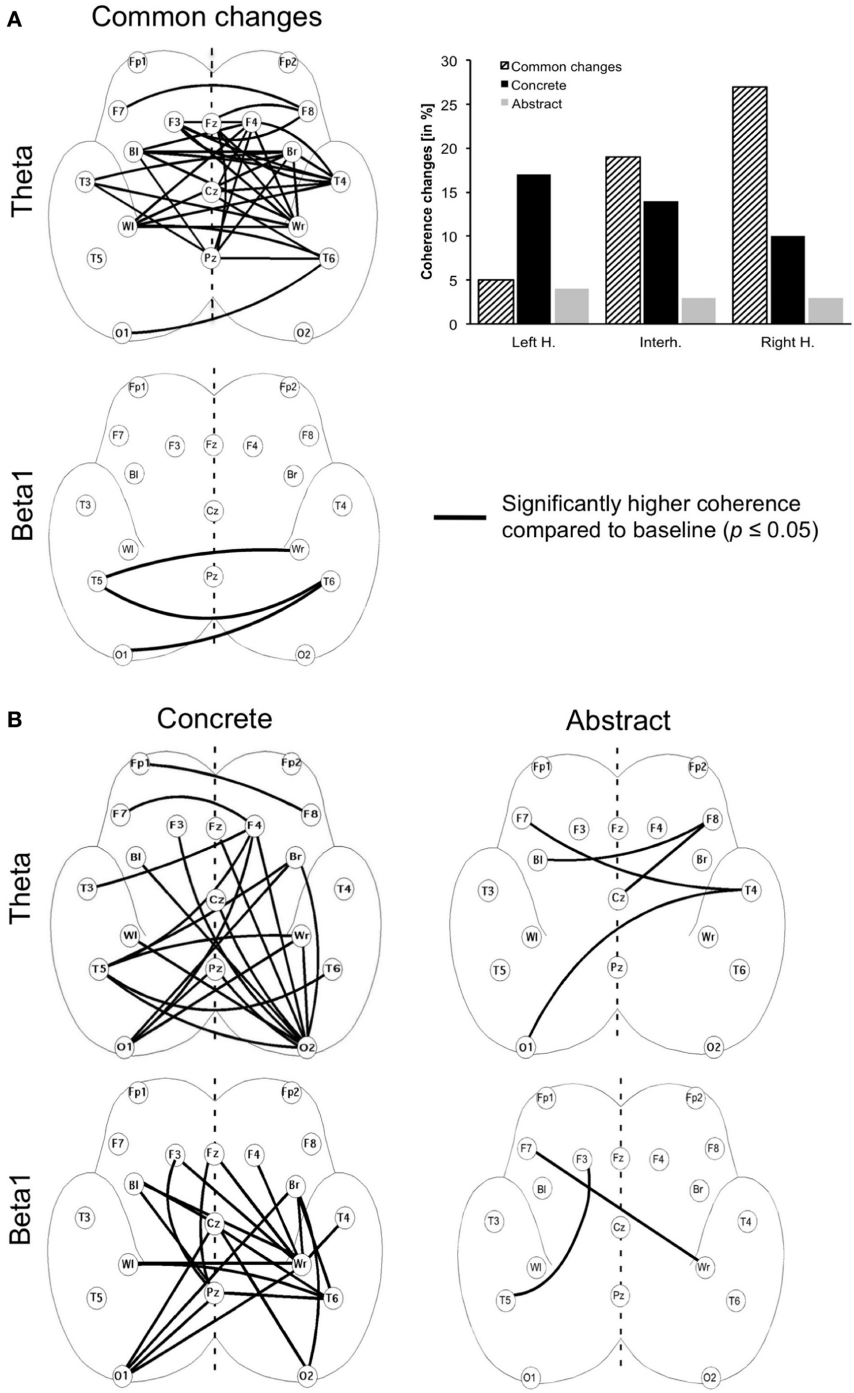
**Long-range coherence changes in the interval 2 (sentence without final noun) compared to the pre-sentence baseline (interval 1).** Error probabilities (*p* = 0.05) are mapped as lines on schematic drawings of the unfolded brain hemispheres. **(A)** Left panel: coherence changes, which occur as well for concrete as for abstract sentences (common changes) for theta and beta1. **(A)** Right panel: percentile coherence changes for left-, right- and interhemispheric electrodes in the theta band. The most common changes are found in the right hemisphere. **(B)** Left panel: coherence increases exclusively associated with the processing of concrete sentences. **(B)** Right panel: coherence increases exclusively associated with the processing of abstract sentences.

To determine which electrode pairs show coherence changes during the processing of concrete or abstract sentences in interval 2, paired Wilcoxon-tests were applied to all possible electrode pairs for theta and beta1 (single coherence analysis). Test results were converted to error probabilities and presented as lines between the electrodes on a schematic drawing of the brain (Figure 4). Normally, with multiple comparisons, significance levels should be adjusted to avoid inflated error probability; however, given the large number of variables (electrodes, coherence values) such adjustments would yield extremely low probabilities for rejecting false null hypotheses. Thus, even real EEG effects might be cancelled out. The statistical procedure therefore, has to be considered as a statistical filter and the error probabilities obtained as purely descriptive, rather than used to confirm or reject the null hypotheses. They are intended to provide indications of probable single coherence differences during the comprehension of concrete and abstract sentences.

Figure 4 shows coherence changes in the interval 2 compared to a pre-sentence baseline of 1000 ms. Strikingly, ~50% of all possible coherence changes in the theta band are found between similar electrodes for both concrete and abstract sentences (Figure 4A, left panel). However, all of these coherence values are higher for the processing of concrete sentences. This means that although both sentences types elicit topographically similar coherence changes, the changes for the concrete sentences are always higher. Paired Wilcoxon-tests showed that 40% of these coherences are significantly higher, even in the single coherence analysis (2*p* = 0.05). Coherence changes between similar electrode pairs for both concrete and abstract sentences are mainly found between temporal, parietal and frontal electrodes as well as central electrodes. In particular, right-hemispheric and interhemispheric electrode pairs exhibit these coherence changes (Figure 4A, right panel). In the beta1 band almost no similar coherence changes are found for concrete and abstract sentences (Figure 4A, left panel).

Apart from these common changes, the processing of concrete sentences was associated with additional specific coherence increases between numerous electrodes in theta as well as beta1 (Figure 4B, left panel). In particular, occipital left- and right-hemispheric and temporo-parietal electrodes show these changes. In theta, left fronto-temporal coherences are also involved. In contrast, processing abstract sentences elicits very few specific coherence increases compared to the baseline (Figure 4B, right panel).

#### Congruence effect at the final noun

**Figure 5:**
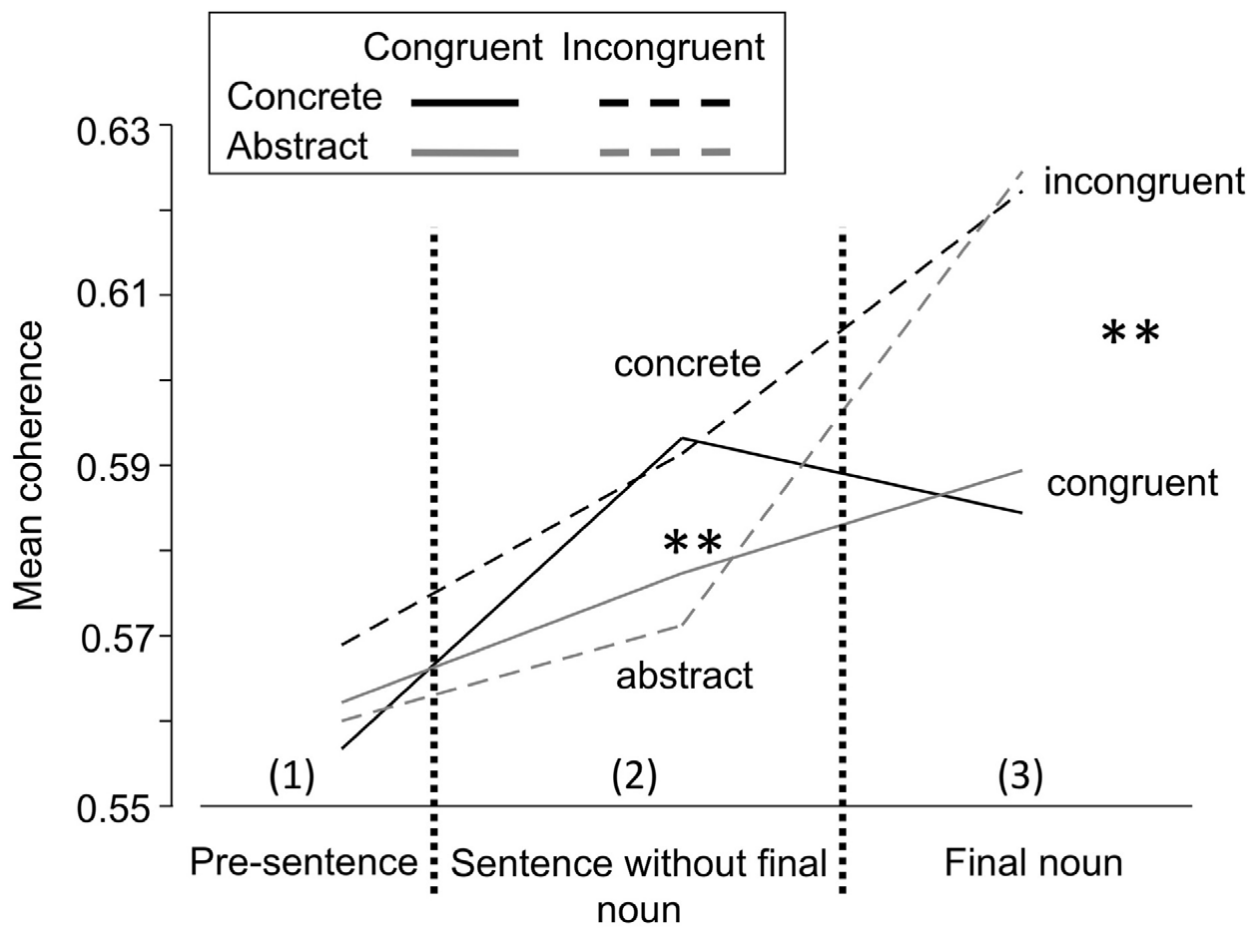
**Effect of the sentences' concreteness and congruence on the mean long-range coherence in the theta frequency band (3–7 Hz) at posterior electrodes.** Whilst processing the first sentence part there is a significant concreteness effect (both black lines vs. both gray lines), whereas a significant congruence effect is found at the final noun (both broken lines vs. both solid lines). Note that one and the same frequency band shows both effects in different sentence intervals. ^**^p < 0.01.

In the sentence interval 3 containing the final noun a strong main effect of congruence on the EEG coherence was found. During the processing of the final noun incongruent sentences elicited a significantly higher coherence than congruent ones, whereby only the theta and gamma2 band showed this effect (congruence × frequency interaction, Table 2). The congruence × frequency × topo-interaction suggests that posterior coherence showed this congruence effect in theta (Figure 5), whereas anterior coherence differed between congruent and incongruent sentences in gamma2. Anterior-posterior coherence showed this effect in both theta and gamma2. Figure 5 shows the dissociation of mean theta coherence at posterior electrodes in the course of the different sentence intervals (Figure 5). The theta band shows a concreteness and congruence effect of sentences in different time intervals.

**Figure 6:**
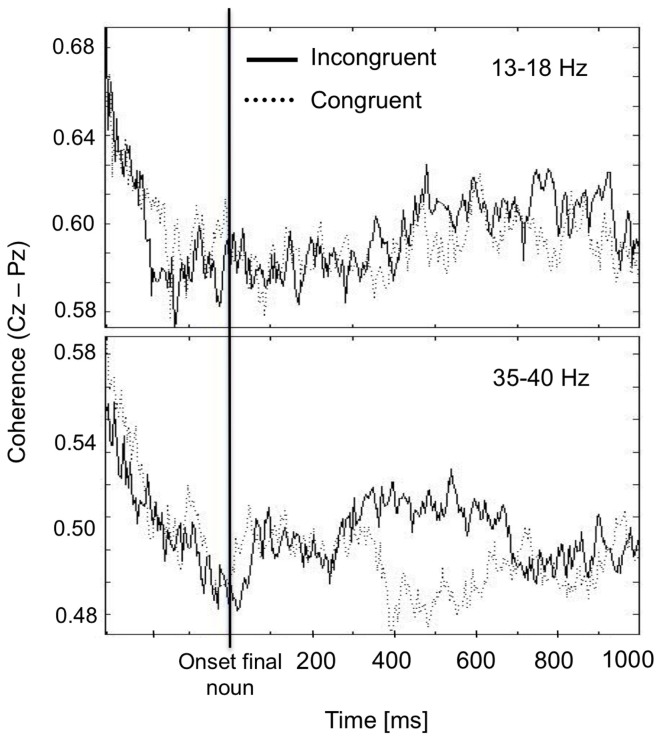
**Difference between the coherence during the processing of congruent and incongruent final nouns.** The gamma2 frequency band (35–40 Hz) shows a higher coherence for the incongruent compared to the congruent sentences from 400 to 600 ms after the final noun's onset. In contrast, the beta1 band does not show this effect.

As stated above, the gamma2 band showed the congruence effect for anterior and anterior-posterior coherences. As an example, Figure 6 illustrates the time-course of mean anterior-posterior coherence (between Cz and Pz) for the beta1 and gamma2 frequency bands for congruent and incongruent final nouns. Whereas the gamma2 band shows a difference between congruent and incongruent nouns in the time window (around 400–600 ms) in which the classical N400 ERP-component is commonly found (Kutas and Hillyard, 1980), the beta1 band did not show such an effect.

**Figure 7:**
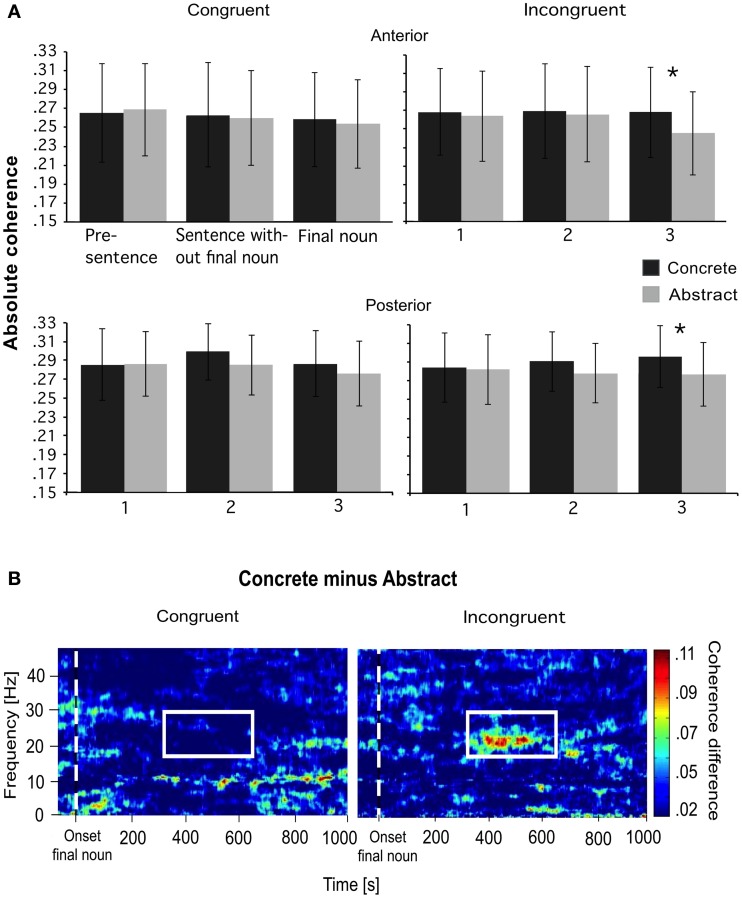
**(A)** Coherence for the congruent and incongruent condition for each sentence interval at anterior and posterior electrodes. Only, the incongruent condition shows significant differences between concrete and abstract sentences in the gamma1 frequency band (interval 3). **(B)** An effect of concreteness on the mean coherence during the final noun for congruent and incongruent sentences (between electrodes Fp1-Bl) for all participants. Significant differences were found for the incongruent condition in the gamma1 band only (from 350 to 650 ms, see white box). ^*^p < 0.05.

An interaction of concreteness × congruence × topo × frequency revealed that the only effect of concreteness on coherence during the processing of the final noun was found in the gamma1 band (29–34 Hz). Analogously as in interval 2, concrete sentences showed higher coherence than abstract ones during the processing of the final noun. However, differences were significant only for the incongruent sentences, not for the congruent ones. The differences were significant at anterior and posterior electrode pairs (Figure 7).

## Discussion

### Concreteness and EEG coherence

During the sentence interval before the final noun (interval 2) there was a strong effect of concreteness on the EEG coherence and, as expected, no congruence effect, since during this interval participants did not know whether a sentence would end congruently or not. In general, concrete sentences elicited significantly more and higher coherence changes than abstract sentences during this sentence interval compared to a pre-sentence baseline. This result was most significant in the theta and beta1 frequency band at posterior sites but was also found at frontal and temporal sites determined by the single coherence analysis.

Corresponding to these results, an increased EEG coherence has frequently been reported for concrete compared to abstract word processing (e.g., Rugg and Venables, 1980; Weiss and Rappelsberger, 1996, 1998; Volf and Razumnikova, 1999; Weiss et al., 2000). With respect to the concept of mental simulations of perception and action during language processing, increased EEG coherence whilst comprehending concrete sentences may reflect the activity of distributed sensoric and motoric brain areas. Thus, the increased coherence between distributed electrodes can be correlated with increased mental simulation of sensoric and motoric events in addition to potential linguistic processes. In particular, visual mental simulation known to be performed in occipito-parietal regions during sentence processing (Just et al., 2004) may be correlated with increased coherence at posterior sites. Also other processes, such as auditory or motoric simulation, may result in higher EEG coherence for concrete language. This finding may be related to a recent study showing that verbs denoting a more concrete motor program (e.g., *to wipe*) show increased brain activity in the inferior parietal lobe compared to verbs denoting a more general motor program (e.g., *to clean*) and abstract verbs (*to ignore*) (van Dam et al., 2010).

In general, the present findings correspond to previous research on EEG coherence and word processing, demonstrating the most reliable word category differences for concrete and abstract nouns in the beta1 frequency band (Weiss and Rappelsberger, 1996). The importance of oscillatory activity in the beta1 band for the question, which of both word categories (concrete or abstract) has been processed, could be supported successfully by machine learning technologies. Thus, analyses with Ordered Means Models (OMMs) succeeded with a probability of ~80% in making a distinction between concrete and abstract words in the beta frequency band (Lingner, 2005). Beta frequencies are also associated with the differentiation between figurative and literal sentences showing increased interhemispheric coherence for the figurative sentences (Berghoff et al., 2005). A recent study on the relationship between the physiology of rhythms and their potential functional roles revealed that beta frequencies are very suitable for coordinating and manipulating multiple cell assemblies which is necessary for higher-order processing (Kopell et al., 2010). Thus, the higher and more widespread coherence for the concrete sentences may indicate the integration of more widespread sensory-motor simulation processes (Weiss and Mueller, 2012).

As mentioned above, processing of concrete sentences correlated with higher posterior coherence not only in the beta1 band but also in theta. A possible explanation for the findings is that theta is also involved in the lexico-semantic retrieval of words serving the binding of different sources of cognitive information for sentence comprehension. Therefore, the posterior coherence network for concrete sentences in theta may indicate a memory-related manipulation of linguistic items beyond the working memory involvement (Bastiaansen et al., 2005; Summerfield and Mangels, 2005).

Another question concerns the kind of neuronal networks participating in the processing of concrete and abstract language, namely do common and/or different synchronization networks exist for both sentence types? Results of this experiment show that many of the coherence changes in the theta frequency band occurred for both the concrete and the abstract sentences. These common networks were found at right temporal and frontal electrodes and between homologue electrodes of the left and right hemisphere. All of these common coherence changes revealed higher values for the processing of concrete sentences. This results in the assumption that the processing of concrete and abstract sentences is based on a topographically comparable synchronization network in theta but possibly puts a quantitatively different demand on this network. The meaning of the above findings can be interpreted considering the topography of these coherence changes and the role the theta and beta bands play in language comprehension. Brain oscillations in the theta range (3–7 Hz) vary with episodic and/or working memory processes (Sarnthein et al., 1998; Klimesch, 1999; Weiss and Rappelsberger, 2000; Bastiaansen et al., 2002; Sauseng et al., 2005; Summerfield and Mangels, 2005; Weiss et al., 2005; Lisman, 2010). Often frontal midline electrodes show the most prominent effects of verbal working memory processes (e.g., Jensen and Tesche, 2002). Therefore, one may argue that the common fronto-central and temporal coherence changes in theta during the semantic analysis of concrete and abstract sentences may correlate with working memory processes. Obviously, both sentence types put certain demands on the working memory system but why is coherence higher and more widespread for concrete sentences? One explanation may be that concrete sentences put a higher demand on the working memory system due to additional visual simulation and more fine-grained mental simulation of other sensory-motor processes.

In the beta1 band there was no common network of coherence changes at all for concrete and abstract sentences. Concrete sentences elicited higher coherences especially at posterior parieto-occipital sites and interhemispherically, which points to an increased interaction between the left and right hemisphere for concrete linguistic material. These findings are in line with an fMRI-study of Just et al. (2004) on high imagery and low imagery sentences, where the active construction of visual images was required. The authors found that both sentence types evoked a common set of cortical regions but high imagery sentences elicited greater overall activation in the brain. Especially the functional connectivity of left parietal regions with core language regions increased during concrete language processing.

Whether concrete and abstract language rely upon a common or different semantic systems has been debated for almost 40 years. Since then, neither the well-known dual-coding theory (Paivio, 1991) nor the context availability theory (e.g., Schwanenflugel and Shoben, 1983), nor the extended versions of the former (Holcomb et al., 1999) or the latter (Levy-Drori and Henik, 2006) on the nature of the concreteness effect could be exclusively confirmed or discarded. In our opinion, the grounding theories postulating the integration of multiple codes (sensory-motor mental simulation) along with experience-based situational context and internal and physical states for both concrete and abstract language combine and extend these older views (e.g., Glenberg and Robertson, 1999; Barsalou, 2008). According to the grounding theories, both common and different neural networks should exist for the processing of concrete and abstract language. The question of which network pattern would be addressed depends on the type of cognitive task.

### Congruence effect at the final noun

A further finding of this experiment concerns the effect of sentence congruence on coherence whilst participants process the final noun of the sentences. Incongruent sentences are associated with higher coherence in the theta and gamma2 band independent of their concreteness. The most significant congruence effect in theta was found mainly at posterior and anterior-posterior sites whereas the congruence effect in gamma2 was mainly found at anterior and anterior-posterior sites. The coherence differences in the gamma2 band were found exactly in the time window in which the N400 usually occurs, namely between 400 and 600 ms after the onset of the final noun.

The well-known N400 ERP component is often interpreted as a measure for reflecting differences in the ease of integration of semantic content showing increased negativity the less an item fits into the given context (Kutas and Hillyard, 1980). Thus, a relation between the N400 component and the finding in the gamma2 band can be assumed (Weiss and Mueller, 2003; Hald et al., 2006). This is underlined by other studies suggesting that gamma oscillations (>30 Hz) correlate with the violation of world-knowledge (Hagoort et al., 2004) and are influenced by semantic integration in sentence processing (Braeutigam et al., 2001; Weiss and Mueller, 2003; Hald et al., 2006). Further, the comparison of correct lexical entries with defect linguistic input was associated with gamma band activity (Pulvermueller et al., 1997; Hannemann et al., 2007). The role of neuronal synchronization in the gamma frequency range, however, is still a matter of debate. Recent studies on the relation of gamma power and semantic violations mostly showed an increased gamma power for congruent sentences (Hald et al., 2006; Penolazzi et al., 2009; Obleser and Kotz, 2011) and also a beta power decrease (16–19 Hz; Wang et al., 2012). Hagoort et al. (2004), however, demonstrated an increase in gamma power only for world-knowledge violations and not for semantic violations.

Considering the different validity of power and coherence measurements (von Stein and Sarnthein, 2000; Weiss and Mueller, 2012), the different frequencies within the gamma band, and tasks investigated in the studies reported above, a final conclusion concerning the behavior of gamma oscillations during the processing of congruent and incongruent sentences cannot be drawn. In the current study, gamma2 band coherence (35–40 Hz) was higher for the incongruent sentences at anterior and anterior-posterior sites, whereas in a previous EEG coherence study gamma coherence around 30 Hz was higher for congruent sentences (Weiss and Mueller, 2003). One reason for these inconsistent results may be that the stimulus material differed considerably between studies, namely simple SVO sentences in the previous study in contrast to the more complex compound sentences in this study. Another reason may be that in the previous study the most significant effect has been found at posterior right-hemispheric sites.

Interestingly, the theta band also revealed a significant congruence effect but this was not restricted to the N400 time window. In contrast, it was present during the whole final noun. Incongruent sentences showed higher coherence especially at posterior and anterior-posterior sites. In this sentence interval, participants had to integrate the final noun into the sentence's context; the theta band may reflect their attempts to do this. Hence, theta activity in this sentence interval may indicate the detection of semantic anomalies or search processes in the sense of lexico-semantic retrieval (Bastiaansen et al., 2002; Hagoort et al., 2004; Davidson and Indefrey, 2007). Power increases in theta have also been assigned to increase with working memory load (Hald et al., 2006) during semantic integration, though these changes were located at more frontal electrodes compared to the present study. The difficulty of integrating an incongruent word into the sentence's context in order to assess whether a sentence is semantically appropriate may be reflected within this frequency band, a result that correlates well with the findings in the gamma1 band.

In the gamma1 band an interaction of concreteness and congruence was found, indicating a coherence difference between concrete and abstract sentences only for the incongruent condition. The interaction of concreteness and congruence in the gamma frequencies is another indication for the prominent role of this band for higher-order cognitive-linguistic processes (Weiss and Mueller, 2012). The gamma1 band is the only frequency band revealing an interaction of concreteness and congruence, particularly at anterior sites but also at posterior ones.

Concrete sentences were associated with higher coherence than abstract sentences from 350 to 650 ms after word onset but only in the incongruent condition. Thus, a supportive sentence context wiped out all the evidence for a concreteness effect at the final noun. The concreteness of the sentences is of less importance when the semantic plausibility of the sentence is high and the meaning is congruent. However, the material's concreteness is much more important when the initial integration fails and, as a consequence, the whole semantic information associated with the final noun becomes potentially relevant. In their attempt to make sense of the sentence, participants try to retrieve and make use of as many mental simulation (e.g., visual simulation at posterior sites) and world-knowledge related processes (at anterior sites; Hagoort et al., 2004) as possible, which probably is easier for concrete sentences. Holcomb et al. (1999) also reported an interaction of congruence and concreteness in the same time window. In their study, the concrete final nouns showed a larger negativity at anterior sites, which is comparable to the findings of the present study. Moreover, the temporal resolution (from 350 to 650 ms) of the concreteness effect at the final noun is comparable to the findings of the study of Holcomb et al. (1999).

In a recent study, it has been shown that gamma power increased only during a violation of world-knowledge in sentences, whereas theta power increased for semantic violation (Hagoort et al., 2004). Other authors, by contrast, reported an increased gamma power for congruent sentences (Hald et al., 2006; Penolazzi et al., 2009; Obleser and Kotz, 2011). This discrepancy in experimental findings could also be based on the unresolved problem that “only an intuitive notion of “semantics,” not grounded in any particular theoretical model” (Pylkkaenen et al., 2011), is often investigated with respect to the neurobiological correlates of sentence-level meaning. Thus, it may be argued that the violation of the concrete sentences in the current study is related to the violation of world-knowledge, whereas the violation of the abstract sentences could be based on a semantic violation in a more restricted sense. Thus, the difference between concrete and abstract final nouns for the incongruent sentences may not base on a concreteness × congruence interaction, but occur due to the different forms of violation. However, we think that in this study incongruent abstract sentences also violate a form of world-knowledge or common ground (Stalnaker, 1978). We only chose those sentences for the EEG-experiment in which at least 80% of the 32 participants inserted the relevant word in a cloze-probability test. Therefore, we suppose that a sort of “common ground” was used by the participants who processed these sentences. These abstract sentences are processed like idioms, which contain words that often occur together (e.g., “Frist setzen”; “set deadline”). Thus, we also think that, during the incongruent abstract sentences, a kind of common ground is violated—not too different from the incongruent, concrete sentences. Therefore, the difference in gamma1 coherence could very well be correlated with a world-knowledge violation in both sentence types, and could thus be correlated with the extinction of the concreteness effect through appropriate context.

## Conclusions

To summarize, our findings on long-range EEG coherence changes indicate that processing of concrete and abstract sentences relies both on common and different neuronal networks. This supports the view proposed by grounding theories, that sensory-motor systems are not only modulated by the comprehension of concrete but also partly of abstract language (e.g., Glenberg et al., 2008; Scorolli et al., 2011). However, concrete language is consistently associated with a higher coherence, proposing the involvement of more neuronal resources for mental sensory-motor simulation processes. It seems as though concrete and abstract language are processed along an analogue continuum and are not strictly separated with respect to their amount and quality of mental simulations. A previous analysis of the height of coherence values during the processing of single sentences and their concreteness/abstractness rating also pointed in this direction. The amount of coherence for a single sentence correlated positively with the offline-rating of concreteness for the respective sentence (Weiss et al., 2009).

The difference of concrete and abstract language was associated with EEG coherence changes within different frequency bands. Whereas the theta band reflected an increased working memory load during sentence processing, in the beta1 band EEG activity was possibly related to different mental simulation processes for concrete and abstract sentences. Activity in the gamma2 band came along with semantic binding processes at the final noun, and the gamma1 band reflected an interaction between concreteness and congruence.

There is no doubt that EEG rhythms are functionally important for cognitive-linguistic processing, but a direct relation between a single frequency and a single cognitive component during language comprehension seems unrealistic (Pulvermueller, 1999; Weiss and Mueller, 2003, 2012; Bastiaansen and Hagoort, 2006). This means that, for example, the 13–18 Hz band supports a certain processing mode of networks involved in distinguishing concrete and abstract language, and may also be involved in other cognitive subprocesses (Weiss and Mueller, 2012). Different EEG rhythms are associated with different components of a cognitive operation, with multiple rhythms potentially playing multiple roles. However, we are still far away from having a complete picture of the meaning of brain oscillations in human language processing, particularly considering non-linear findings and relations between different frequencies at the 1:1 or m:n level (Schack and Weiss, 2005). Nevertheless, research on brain oscillations during comprehension of concrete and abstract language reveals new findings on the neurobiological dynamics underlying language comprehension and adds new aspects to the findings revealed with other electrophysiological or hemodynamic methods.

### Conflict of interest statement

The authors declare that the research was conducted in the absence of any commercial or financial relationships that could be construed as a potential conflict of interest.
